# Case Report: A case of carbapenem hypersensitivity and tigecycline-associated coagulopathy after immunotherapy for advanced oral cancer

**DOI:** 10.3389/fmed.2026.1863232

**Published:** 2026-06-30

**Authors:** Yifei Peng, Rui Peng

**Affiliations:** Leshan Hospital of Traditional Chinese Medicine, Affiliated to Chengdu University of Traditional Chinese Medicine, Leshan, Sichuan, China

**Keywords:** carbapenem allergy, clinical pharmacist, coagulopathy, immunotherapy, oral squamous cell carcinoma, sepsis, tigecycline

## Abstract

**Background:**

Managing complex infections after immunotherapy in patients with advanced head and neck cancer is clinically challenging, especially when severe adverse drug reactions like carbapenem hypersensitivity and tigecycline-associated coagulopathy further limit treatment options. This case demonstrates the potential contribution of clinical pharmacists in such difficult scenarios.

**Case presentation:**

A 74-year-old man with oral squamous cell carcinoma developed severe sepsis after one cycle of PD-1 inhibitor (penpulimab) plus EGFR inhibitor (cetuximab). The clinical pharmacist sequentially recommended escalation to meropenem, switch to piperacillin-tazobactam, and addition of vancomycin. Later, the pharmacist recognized possible imipenem-related central nervous system effects, leading to discontinuation of all carbapenems due to possible cross-hypersensitivity. Subsequently, tigecycline was initiated, its associated coagulopathy was managed, and a switch to doxycycline was made.

**Results:**

The patient’s fever resolved, and coagulation substantially improved; the infection was finally controlled. Causality was assessed using the Naranjo scale (imipenem 7, meropenem 5) and the RUCAM scale (penpulimab 6).

**Conclusion:**

In this case, the clinical pharmacist’s dynamic assessment and use of quantitative adverse reaction tools (Naranjo and RUCAM scales) contributed to successful infection control and management of drug toxicity.

## 1 Introduction

Programmed cell death protein 1 (PD-1) inhibitors may cause immune-related adverse events, while epidermal growth factor receptor (EGFR) inhibitors disrupt the skin and mucosal barrier, increasing the risk of bacterial invasion (1, 2). When both agents are used, patients may develop complex infections along with complications such as drug-induced liver injury. In hospitalized immunocompromised patients, these infections often involve multidrug-resistant organisms, and management principles align with guidelines for nosocomial pneumonia (3, 4). When multidrug-resistant bacteria are involved, or when adverse reactions leave no suitable antimicrobial option remaining, clinical management becomes a clinically challenging scenario.

This case report describes a patient with advanced oral cancer who developed severe sepsis after PD-1/EGFR inhibitor therapy, followed by suspected carbapenem hypersensitivity and tigecycline-associated coagulopathy. The clinical pharmacist participated in stepwise optimization of the anti-infective regimen and identification of adverse reactions. The aim is to provide a reference for managing similar complex scenarios.

## 2 Case report

### 2.1 Patient presentation

A 74-year-old man (height 165 cm, weight 55 kg) was admitted to our hospital on December 31, 2025. He had a 7-month history of recurrent left-sided gingival pain. One week after receiving targeted therapy for oral cancer, he developed worsening fever that had lasted for 3 days.

In July 2025, the patient underwent a tooth extraction at an outside hospital for left-sided gum pain, but the pain did not resolve. In October 2025, a left palatal mass was found, and biopsy confirmed squamous cell carcinoma. In November 2025, a mass appeared on the right side of the neck. To treat the oral cancer, he received one cycle of combination immunotherapy on December 26, 2025, consisting of penpulimab (PD-1 inhibitor, 200 mg) plus cetuximab (EGFR inhibitor, 500 mg). One week later, on December 31, he developed a high fever (39.3 °C) and was admitted to our hospital with altered mental status.

*Past history*: generally healthy; no history of hypertension, diabetes, drug or food allergies.

*Physical examination*: T 39.3 °C, P 121 beats/min, respiratory rate 22/min, BP 109/65 mmHg. Malnourished, chronic disease appearance. A fixed, non-tender lymph node about the size of an egg was palpable on the right side of the neck. Symmetrical 2 + pitting edema of both lower limbs.

### 2.2 Laboratory findings on admission

On admission, the white blood cell (WBC) count was 17.54 × 10^9^ /L with 92.4% neutrophils. C-reactive protein (CRP) was 139.73 mg/L, procalcitonin (PCT) 15.86 ng/mL, and interleukin-6 (IL-6) 1424.29 pg./mL. Albumin was 33.4 g/L, aspartate aminotransferase (AST) 64.6 U/L, and D-dimer 7.78 μg/mL. Chest and neck CT showed left maxillary bone destruction with a soft-tissue mass, cervical lymph node metastases, and bilateral pulmonary edema.

*Admission diagnoses*: 1. Oral squamous cell carcinoma with cervical lymph node metastasis (T4bN3bM0, stage IVb, PD-L1 CPS = 80) after targeted therapy 2. Oral soft tissue infection 3. Severe malnutrition.

### 2.3 Intervention and clinical course

**Figure 1:**
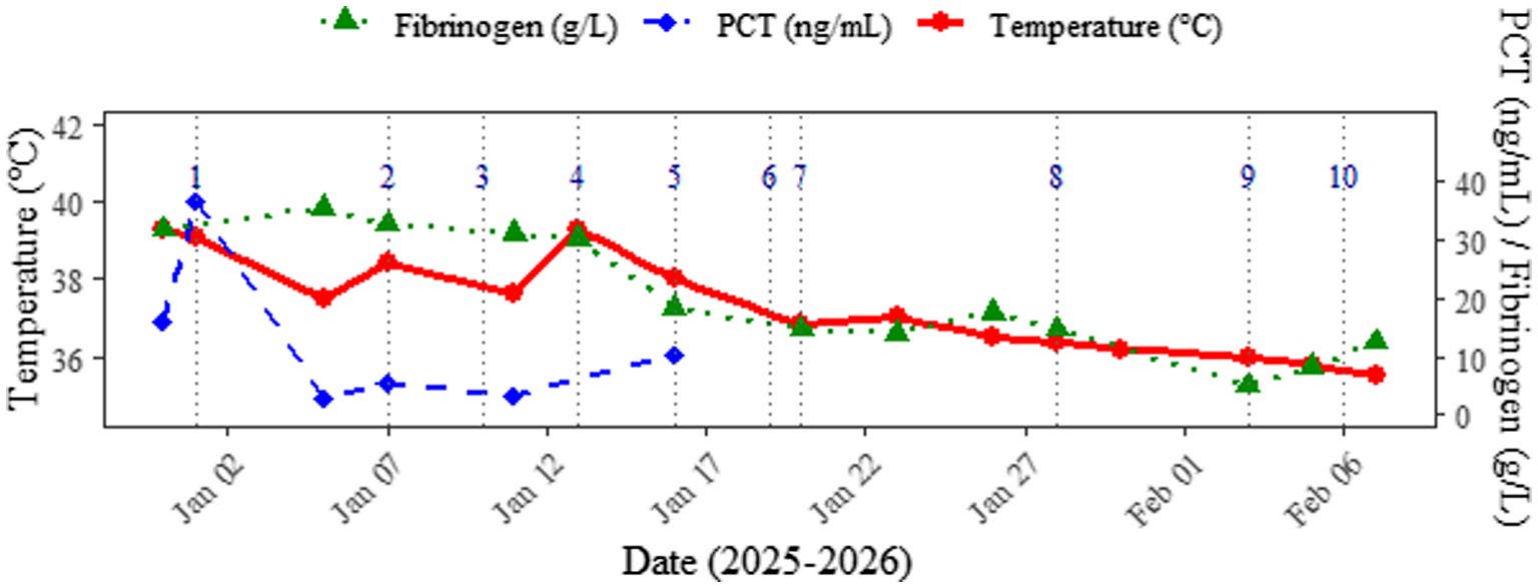
Clinical course and antibiotic adjustments over time: temperature, PCT, fibrinogen and key interventions. Events: 1 = Start Meropenem; 2 = De-escalation; 3 = Add Vancomycin; 4 = Start Imipenem; 5 = Stop Imipenem, back to Meropenem; 6 = Stop Carbapenems (allergy); 7 = Vancomycin + Ceftazidime; 8 = Start Tigecycline; 9 = Stop Tigecycline (coagulopathy); 10 = Start Doxycycline.

The treatment course was complex and included several phases. The clinical pharmacist participated throughout. Figure 1 illustrates the entire clinical course, including temperature, PCT, fibrinogen, and key medication adjustments.

#### 2.3.1 Phase 1 (December 31–January 6)

The patient presented with severe sepsis (meeting Sepsis-3 criteria: infection plus an acute increase in SOFA score of at least 2 points, as evidenced by altered mental status and tachypnea) with acute left heart failure. Initial regimen was piperacillin-tazobactam (4.5 g q8h). The next day (January 1), infection markers worsened: WBC 21.20 × 10^9^ /L, PCT 36.73 ng/mL, IL-6117.39 pg./mL. The pharmacist recommended upgrading to meropenem (1 g q8h) plus intensive fluid management and diuretics, because the initial regimen failed to control the infection and the patient was at high risk for drug-resistant gram-negative bacteria. After switching to meropenem, the patient’s temperature returned to normal by January 5. On January 6, PCT decreased to 2.67 ng/mL (from 36.73 ng/mL on January 1) and IL-6 to 58.93 pg./mL (from 117.39 pg./mL on January 1).

#### 2.3.2 Phase 2 (January 7–16)

On January 7, the patient developed a recurrent fever (38.4 °C) and white plaques on the oral mucosa. The pharmacist considered possible causes including uncontrolled infection, drug fever, and fungal superinfection. The patient had no history of liver disease, and no other hepatotoxic medications were used. The Naranjo scale (5) gave a score of 1–2 for meropenem-associated liver injury (“possible”), while the RUCAM scale (6) gave a score of 6 for penpulimab (“probable”). Given the temporal relationship and the known hepatotoxicity of PD-1 inhibitors, the liver injury was considered more likely related to the PD-1 inhibitor, although other contributing factors cannot be entirely excluded. We acknowledge that RUCAM has limitations in complex, polymedicated patients and should be interpreted with caution. The pharmacist recommended step-down to piperacillin-tazobactam (4.5 g q8h) plus sodium bicarbonate gargle and topical nystatin. On January 10, liver enzyme levels decreased: ALT from 127.4 U/L to 60.2 U/L, and AST from 84.6 U/L to 45.1 U/L.

**Figure 2:**
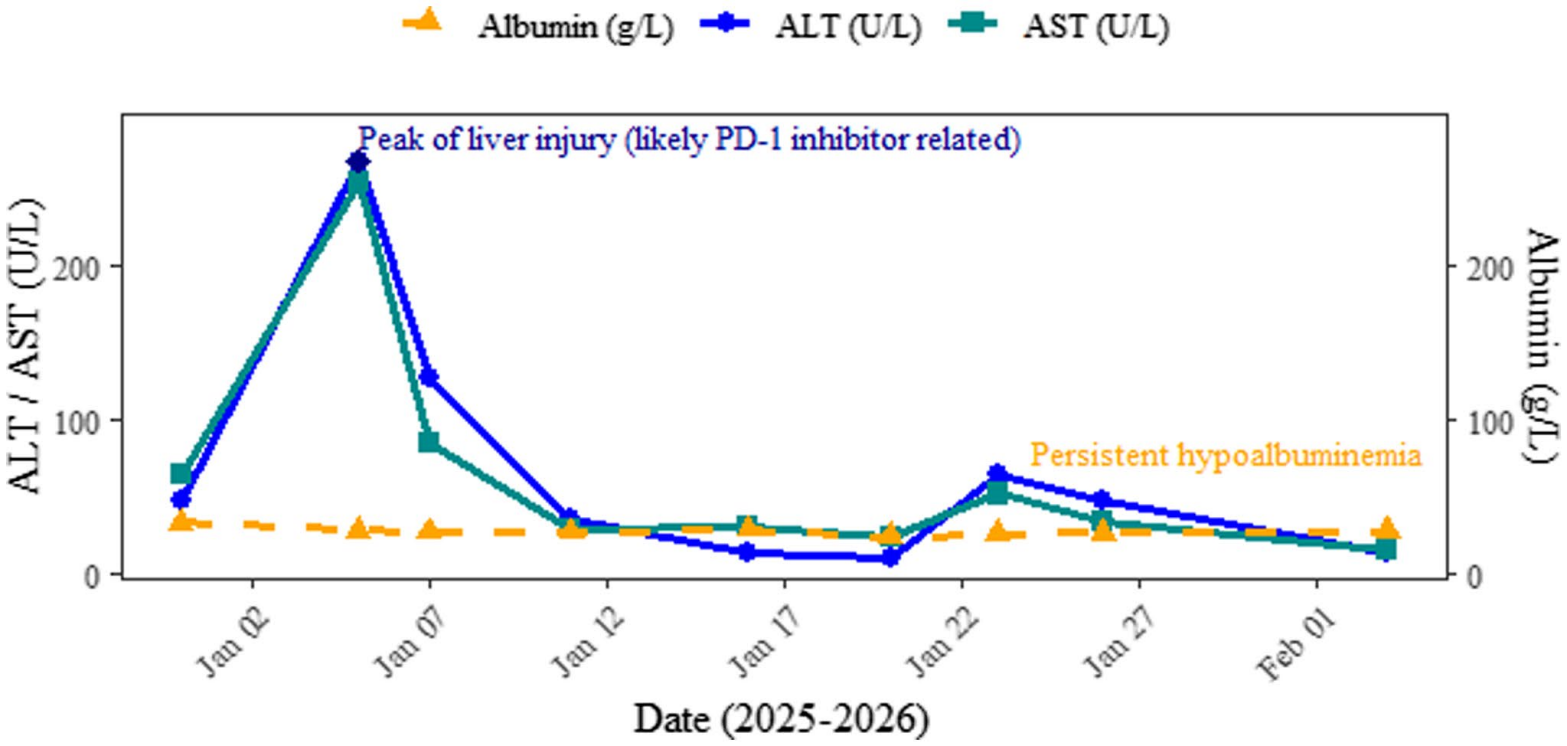
Liver function and albumin dynamics: ALT, AST and albumin levels over time. The peak of liver injury (likely PD 1 inhibitor related) is indicated.

**Figure 3:**
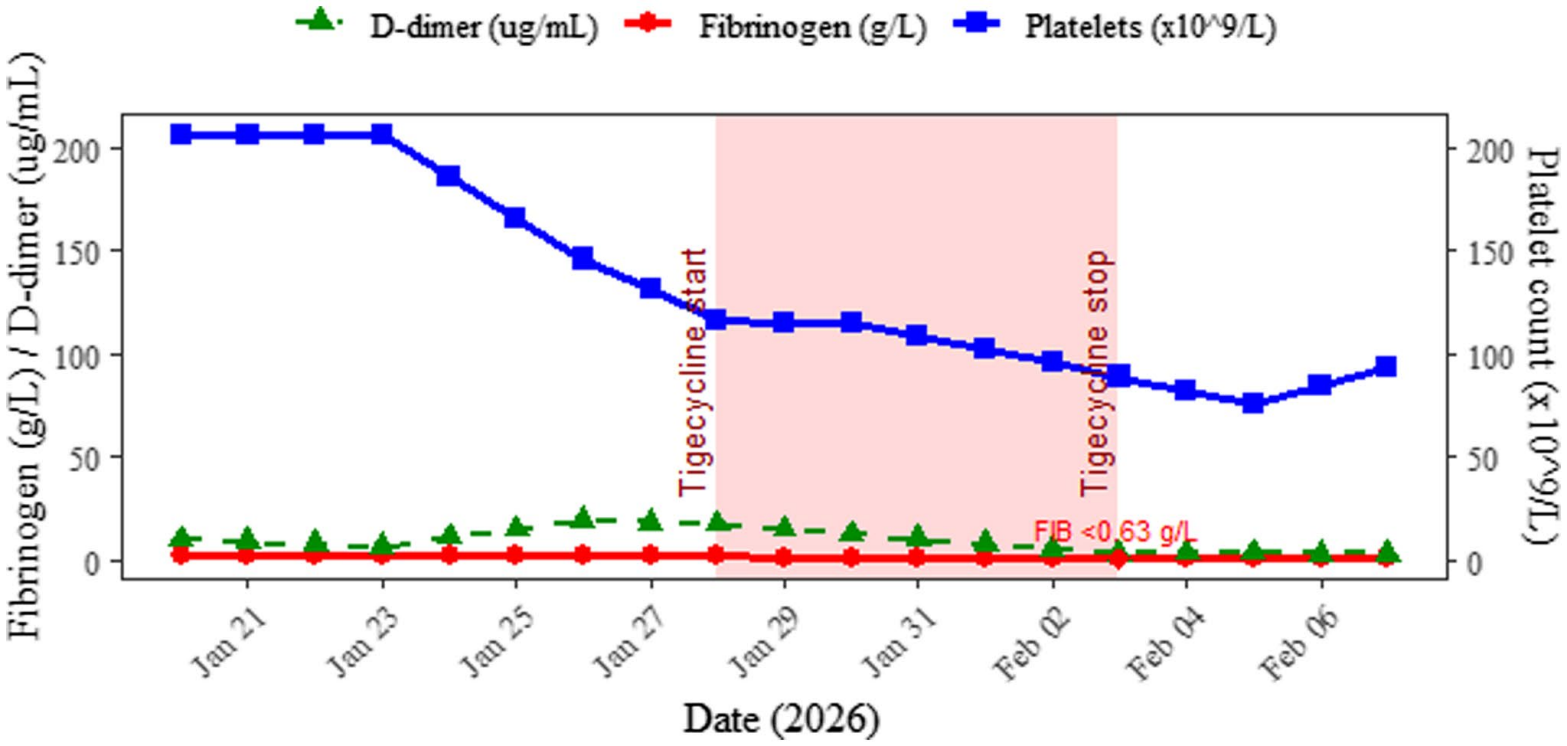
Tigecycline associated coagulopathy: dynamic changes of fibrinogen, platelets and D dimer (μg/mL).

However, after step-down the patient again spiked fever (39.3 °C) with infection markers that rose again: PCT increased to 5.26 ng/mL (from 2.67 ng/mL on January 6) and IL-6 increased to 494.27 pg./mL (from 58.93 pg./mL on January 6). Oral secretion culture grew *Enterococcus faecalis* and methicillin-resistant coagulase-negative *Staphylococcus epidermidis* (MRCNS). These organisms were considered likely oral colonizers rather than the primary cause of sepsis, because multiple microorganisms were present simultaneously, blood cultures remained negative, and the persistently high PCT level was more consistent with gram-negative infection. However, their potential contribution to the infectious process cannot be completely excluded. Vancomycin (1 g q12h, adjusted for renal function) was added. On January 13, D-dimer rose to 29.36 μg/mL and infection indicators continued to rise, so imipenem/cilastatin (1 g q8h) was started while discontinuing the prior two drugs. On January 16, the patient developed a generalized rash, hyperarousal, and incoherent speech. The Naranjo score (5) for imipenem was 7 (“probable”). Imipenem was stopped and meropenem restarted. The psychiatric symptoms resolved completely within 48 h after discontinuation, and the rash gradually subsided over the following days. Septic encephalopathy, metabolic disturbances, and hypoxia were ruled out based on normal electrolytes, stable blood glucose, adequate oxygenation, and absence of acute brain lesions on CT (see Figures 2, 3).

#### 2.3.3 Phase 3 (January 17–27)

After switching back to meropenem, the patient’s temperature normalized briefly, but on January 19 he developed large erythematous, wheal-like lesions with severe itching. The Naranjo score (5) for meropenem rash was 5 (“probable”); carbapenem cross-hypersensitivity was suspected. All carbapenems were discontinued, and antihistamines plus topical corticosteroids were given. The patient’s creatinine clearance (Ccr) was 36.51 mL/min. Based on the vancomycin consensus guideline (7), a reduced maintenance dose of vancomycin (1 g q24h) was selected to avoid accumulation and nephrotoxicity, and ceftazidime (2 g q12h) was added for broader gram-negative coverage. However, after 4 days (January 26), infection markers increased sharply again: PCT 10.29 ng/mL and IL-6204.83 pg./mL.

During a multidisciplinary discussion on January 28, the pharmacist noted that the persistently high PCT level (>10 ng/mL) raised suspicion of a gram-negative bacillus infection. Given the patient’s prolonged hospitalization and prior broad-spectrum antibiotic exposure, carbapenem-resistant *Acinetobacter baumannii* (CRAB) was considered a possible pathogen, although no microbiological confirmation was available. Tigecycline carries a warning of increased all-cause mortality (8). The patient also had severe hypoalbuminemia (Alb 26.5 g/L), which may have increased the risk of coagulopathy and gastrointestinal adverse effects (9, 10). However, because other antimicrobial options had failed or were not tolerated (the patient had already failed piperacillin-tazobactam, vancomycin + ceftazidime, and carbapenems were contraindicated due to suspected cross-reactivity), the benefits of tigecycline were considered to outweigh the potential risks. The concentration of tigecycline in lung epithelial lining fluid is approximately 20% of the blood concentration (11); therefore, lung imaging was closely monitored. The patient received a loading dose of 100 mg of tigecycline (the recommended loading dose is 200 mg, but this was not available at our hospital), followed by 50 mg every 12 h.

#### 2.3.4 Phase 4 (January 28–February 5)

After tigecycline was started, temperature normalized within 3 days. On January 31, IL-6 decreased to 9.63 pg./mL; on February 3, PCT decreased to 5.06 ng/mL and CRP to 17.25 mg/L. The infection was finally controlled.

On February 3 (day 7 of tigecycline), severe laboratory abnormalities were observed: fibrinogen <0.63 g/L and platelet count 89 × 10^9^ /L. The pharmacist suspected tigecycline-associated coagulopathy. Coagulation factors II, VII, IX, and X were normal, and no schistocytes were seen on peripheral blood smear, making disseminated intravascular coagulation unlikely. Sepsis-associated coagulopathy was also considered unlikely because the patient’s infection markers had been improving and the coagulopathy resolved rapidly after tigecycline withdrawal without specific anti-DIC therapy. Vitamin K1 is not reliably effective for this condition (12). Tigecycline was immediately stopped, and fresh frozen plasma and human fibrinogen were transfused. Because the infection had been largely controlled (temperature normal, PCT decreased to 5.06 ng/mL) and continued intravenous treatment was no longer necessary, a switch to oral doxycycline (100 mg bid) was made for step-down maintenance therapy, despite its lower activity against CRAB compared to tigecycline. On February 6, fibrinogen rose to 1.40 g/L and platelets to 80 × 10^9^ /L; on February 8, fibrinogen reached 1.9 g/L. Coagulation function improved significantly, and the patient had no further fever or bleeding.

### 2.4 Outcome

The patient was discharged after a prolonged hospital stay. At discharge, he was afebrile, hemodynamically stable, and able to eat orally. Coagulation parameters had substantially improved (fibrinogen 1.9 g/L, platelets 94 × 10^9^ /L), and liver function tests were within normal limits. No further anti-infective therapy was required.

## 3 Discussion

This case highlights several clinically challenging aspects of managing post-immunotherapy infections: the difficulty of identifying pathogens without positive cultures, the risk of carbapenem cross-hypersensitivity, and the occurrence of tigecycline-associated coagulopathy in a patient with hypoalbuminemia. We summarize the following practical lessons (see Table 1).

**Table 1:** Summary of key pathogen and immunological findings.

Test category	Test item	Main results	Clinical significance
Blood culture	Aerobic/anaerobic dual bottles (multiple times)	Multiple submissions (Jan 5, Jan 13, Jan 25, etc.) all showed “no bacterial growth after 5 days”	No etiological evidence obtained, supporting the limitation statement of “lack of positive blood culture results” in the article
Oral secretion culture	Routine bacterial culture (Jan 7)	*Enterococcus faecalis*, *Staphylococcus epidermidis* (MRCNS, methicillin-resistant coagulase-negative staphylococcus)	Suggests oral colonizers, but not the definite pathogen of bloodstream infection
Sputum culture	Routine bacterial culture (Jan 25)	Pure culture of *Enterococcus faecalis* (4+), drug susceptibility showed sensitivity to ampicillin, vancomycin, and linezolid	Further supports enterococci as predominant colonizers, but markedly elevated PCT is not consistent with enterococcal bloodstream infection
Mixed growth from secretions	Routine bacterial culture (Jan 21)	“Three or more microorganisms including *Enterococcus faecalis*, *Enterococcus faecium*, and *Staphylococcus epidermidis*”	Mixed colonization of multiple microorganisms, difficult to determine the true pathogen
Fungal workup	G test (Jan 28)	<37.50 pg./mL (reference <95)	Negative, does not support invasive fungal infection
	GM test (Jan 28)	0.11 S/CO (reference <0.5)	Negative, does not support Aspergillus infection
	Sputum Gram stain (Jan 25)	Yeast-like fungal spores seen (3+)	Likely colonization or contamination; combined with negative G/GM tests, antifungal therapy not required
Immune status	T cell subsets (Jan 16)	CD3 + T 644/μL (↓), CD4 + T 416/μL (↓), CD8 + T 224/μL (↓), CD4/CD8 = 1.86	Cellular immunosuppression, consistent with immune activation but lymphopenia following PD-1 inhibitor use
	Antinuclear antibody (ANA)	Positive (1:100 nuclear granular + 1:100 nuclear membranous pattern), anti-dsDNA and anti-ENA profile negative	Low-titer positivity, no evidence of autoimmune disease
	Antineutrophil cytoplasmic antibody (ANCA)	Anti-MPO, anti-PR3, anti-GBM all negative	Excludes vasculitis-related diseases
Other infection markers	T-SPOT. TB (Jan 29)	TB-IGRA (T-N) = 0.46 pg./mL (<14), result interpreted as “negative”	Excludes tuberculosis infection
	Influenza A/B antigens (Jan 16, Jan 13)	Both negative	Excludes influenza virus infection

### 3.1 Pathogen inference without positive cultures

The absence of positive blood cultures was a major limitation. Multiple cultures were obtained after antibiotics had already been started, which may have reduced sensitivity; additionally, fastidious organisms or prior partial treatment can contribute to culture negativity. Therefore, the pharmacist relied on clinical judgment and biomarker trends rather than microbiological confirmation. The persistently elevated PCT (>10 ng/mL) and rising IL-6 suggested an ongoing gram-negative infection, but these markers are not pathogen-specific and should be interpreted as supportive, not definitive (13). Based on the patient’s prolonged hospitalization and carbapenem exposure, carbapenem-resistant *Acinetobacter baumannii* (CRAB) was considered a possible pathogen; this was an empirical inference, not a microbiologically proven diagnosis. Such “phenotype-driven” reasoning can be useful in resource-limited settings, but it must be distinguished from culture-based or molecular diagnostics. Whenever possible, metagenomic next-generation sequencing (mNGS) should be pursued to improve diagnostic certainty (14, 15).

### 3.2 Carbapenem cross-hypersensitivity and Naranjo score

The patient developed rash and psychiatric symptoms to both imipenem and meropenem, with a positive rechallenge to meropenem, which strongly suggests cross-reactivity. The reported cross-reactivity rate among carbapenems can be as high as 47% in some studies (16, 17); however, these estimates are largely derived from patients with known penicillin allergy, and the true rate in patients without prior *β*-lactam allergy is less certain. Therefore, our observation is consistent with, but does not fully confirm, cross-hypersensitivity in the absence of formal allergy testing.

Using the Naranjo scale (5), we obtained scores of 7 for imipenem and 5 for meropenem (“probable”). While the Naranjo scale provides a structured approach to causality assessment, it has limitations in complex, real-world scenarios where multiple comorbidities and concomitant medications are present. The scores should be interpreted as supportive evidence rather than definitive proof. Nonetheless, they offered a useful quantitative framework for clinical decision-making in this case. Table 2 presents the causality assessment for all major adverse drug reactions.

**Table 2:** Causality assessment of major adverse drug reactions.

Suspected drug	Adverse reaction	Assessment scale	Scoring items and scores	Total score	Causality grade	Management and outcome
Imipenem/cilastatin	Psychiatric symptoms (confusion, agitation) + generalized rash	Naranjo scale	① Reaction occurred after drug administration (+2); ② Symptoms improved after drug discontinuation (+1); ③ Drug known to cause CNS adverse reactions (+1); ④ No similar symptoms with prior meropenem use (+1); ⑤ Other causes excluded (intracranial infection, electrolyte disorders, etc.) (+2)	7	Probable	Psychiatric symptoms resolved within 48 h after discontinuation; rash gradually subsided
Meropenem	Generalized erythema and urticarial rash (positive rechallenge)	Naranjo scale	① Positive rechallenge (+3); ② Symptoms improved after drug discontinuation (+1); ③ Drug known to cause allergy (+1)	5	Probable	Symptoms resolved after discontinuation and antihistamine/topical corticosteroid therapy; all carbapenems contraindicated thereafter
Penpulimab (PD-1 inhibitor)	Drug-induced liver injury (ALT 267.8 U/L, AST 254.9 U/L)	RUCAM scale	① Time to onset 5–90 days (+2); ② ALT decreased >50% after drug discontinuation (+2); ③ No rechallenge (0); ④ Other causes excluded (+1); ⑤ Drug known to have hepatotoxicity reports (+1)	6	Probable	Liver function normalized after hepatoprotective therapy; PD-1 inhibitor not reused
Tigecycline	Severe coagulopathy (fibrinogen <0.63 g/L, platelet count decreased to 89 × 10^9^/L)	Clinical judgment + temporal association	① Occurred on day 7 of therapy; ② Resolved after drug discontinuation and plasma/fibrinogen infusion; ③ Tigecycline known to cause hypofibrinogenemia (12, 13); ④ Hypoalbuminemia (Alb 26.5 g/L) as risk factor	—	Probable	Immediate discontinuation, infusion of fresh frozen plasma and human fibrinogen, switch to doxycycline; coagulation function gradually recovered

### 3.3 Tigecycline-associated coagulopathy and hypoalbuminemia

#### 3.3.1 Literature summary

The incidence of tigecycline-induced coagulopathy is approximately 2–5%, with risk factors including treatment duration >5 days, hypoalbuminemia, and renal insufficiency (18–20). A 2025 retrospective study (*n* = 114) found that tigecycline exposure (AUC_0-24_) was highly predictive of hypofibrinogenemia, with a recommended threshold of 17.03 mg·h/L (21). Another study confirmed total dose as an independent risk factor (OR = 15.28, *p* = 0.01) and baseline fibrinogen as a protective factor (OR = 0.53, *p* = 0.04) (18). These data suggest that cumulative tigecycline dose strongly predicts coagulopathy, whereas higher baseline fibrinogen is protective. Thus, monitoring cumulative dose and baseline fibrinogen levels can help identify patients at increased risk.

#### 3.3.2 Patient-specific interpretation

In our patient, albumin was 26.5 g/L, and severe hypofibrinogenemia (<0.63 g/L) developed on day 7 of tigecycline therapy. Tigecycline is approximately 80% protein-bound; hypoalbuminemia increases free drug concentration, volume of distribution, and possibly clearance, thereby raising coagulopathy risk (9, 10). The pharmacist promptly discontinued tigecycline, administered plasma and fibrinogen, and avoided ineffective vitamin K1 (12). Coagulation parameters recovered after switching to doxycycline.

### 3.4 Implications for clinical pharmacy practice

#### 3.4.1 Based on this case, we recommend

Immunosuppressed host sepsis: In this case, after failure of initial empiric therapy, escalation to a carbapenem was a reasonable choice. However, the decision to escalate should always balance the risk of resistance induction against the patient’s clinical condition, and this strategy may not be applicable to all settings (22).

#### 3.4.2 Adverse reaction identification

In this case, the combined use of the Naranjo (5) and RUCAM (6) scales provided a structured framework for causality assessment. Whether this approach may improve attribution accuracy in broader clinical settings requires further validation.

#### 3.4.3 Tigecycline safety

Based on this case and the supporting literature (9, 10, 21), frequent monitoring of coagulation (e.g., at least twice weekly) may be considered after day 5, especially in patients with hypoalbuminemia. However, this recommendation should be interpreted cautiously as it is derived from a single case experience.

#### 3.4.4 Alternative treatment

In this case, because tigecycline had to be discontinued due to coagulopathy and the infection was largely controlled (afebrile, PCT decreased), doxycycline was chosen for oral step-down maintenance therapy. Its activity against CRAB is generally lower than that of tigecycline (23), and other potential alternatives (e.g., minocycline) were not available. This approach should be reserved for patients who have already shown significant clinical improvement, and it requires careful follow-up. It may not be suitable for patients with unresolved infection or those needing continued intensive therapy.

### 3.5 Limitations

This single-center case has several limitations. First, no microbiological confirmation was obtained (no mNGS, negative blood cultures); tigecycline was initiated based on clinical inference rather than susceptibility testing. Second, vancomycin therapeutic drug monitoring was unavailable, so dosing relied on estimated renal function (7). Third, the tigecycline loading dose was only 100 mg instead of the recommended 200 mg, which may have delayed early efficacy (9). These limitations should be considered when interpreting the findings.

## 4 Conclusion

This case illustrates that dynamic assessment, quantitative adverse reaction tools, and comprehensive comparison of alternatives can help clinical pharmacists and physicians find a balance between anti-infection and safety in complex clinical scenarios. The findings suggest the potential value of multidisciplinary pharmacologic input in challenging antimicrobial decision-making, although further studies are needed to generalize these observations.

## Data Availability

The original contributions presented in the study are included in the article/supplementary material, further inquiries can be directed to the corresponding author.

## References

[1] Schneider BJ Naidoo J Santomasso BD Lacchetti C Adkins S Anadkat M . Management of immune-related adverse events in patients treated with immune checkpoint inhibitor therapy: ASCO guideline update. J Clin Oncol. (2021) 39:4073–126. doi: 10.1200/JCO.21.01440, 34724392

[2] Thompson JA Schneider BJ Brahmer J Achufusi A Armand P Berkenstock MK . Management of immunotherapy-related toxicities, version 1.2022, NCCN clinical practice guidelines in oncology. J Natl Compr Cancer Netw. (2022) 20:387–405. doi: 10.6004/jnccn.2022.0020, 35390769

[3] Martin-Loeches I Torres A Nagavci B Aliberti S Antonelli M Bassetti M . ERS/ESICM/ESCMID/ALAT guidelines for the management of severe community-acquired pneumonia. Intensive Care Med. (2023) 49:615–632. doi: 10.1007/s00134-023-07033-8 37012484 PMC10069946

[4] Metersky ML Kalil AC Klompas M Muscedere J Sweeney DA Palmer LB . Executive summary: management of adults with hospital-acquired and ventilator-associated pneumonia: 2016 clinical practice guidelines by the Infectious Diseases Society of America and the American Thoracic Society. Clin Infect Dis. (2016) 63:575–82. doi: 10.1093/cid/ciw504, 27521441 PMC4981763

[5] Naranjo CA Busto U Sellers EM Sandor P Ruiz I Roberts EA . A method for estimating the probability of adverse drug reactions. Clin Pharmacol Ther. (1981) 30:239–45. doi: 10.1038/clpt.1981.154, 7249508

[6] Danan G Teschke R. RUCAM in drug and herb induced liver injury: the update. Int J Mol Sci. (2015) 17:14. doi: 10.3390/ijms17010014, 26712744 PMC4730261

[7] Rybak MJ Le J Lodise TP Levine DP Bradley JS Liu C . Therapeutic monitoring of vancomycin for serious methicillin-resistant *Staphylococcus aureus* infections: a revised consensus guideline and review by the American Society of Health-System Pharmacists, the Infectious Diseases Society of America, the Pediatric Infectious Diseases Society, and the Society of Infectious Diseases Pharmacists. Am J Health Syst Pharm. (2020) 77:835–64. doi: 10.1093/ajhp/zxaa036, 32191793

[8] He H Zheng Y Liu J Akine D Sasahara T Koido A . Case of a pregnant woman with probable prolonged SARS-CoV-2 viral shedding 221 days after diagnosis. J Infect Chemother. (2022) 28:998–1000. doi: 10.1016/j.jiac.2022.03.012, 35367149 PMC8958094

[9] Su W Song S Liu J Yu H Feng B Wu Y . Population pharmacokinetics and individualized dosing of tigecycline for critically ill patients: a prospective study with intensive sampling. Front Pharmacol. (2024) 15:1342947. doi: 10.3389/fphar.2024.1342947, 38348395 PMC10859475

[10] Dai A Zheng F Liu J Chen Q Zhou Z Yu L . Population pharmacokinetics of tigecycline and implications for individualized therapy optimization: a systematic review. Drug Des Devel Ther. (2025) 19:10045–60. doi: 10.2147/DDDT.S553622, 41244714 PMC12619605

[11] Russo A Taccari F Di Paolo A Misawa K Nishimura T Yoshikawa M . Combined dual β-lactams and diazabicyclooctane β-lactamase inhibitor is highly effective against *Mycobacterium abscessus* species in vitro. J Glob Antimicrob Resist. (2025) 42:142–50. doi: 10.1016/j.jgar.2025.02.015, 40015478

[12] Sun H Meng X Shao X Duan L Fan K. Impact of tigecycline on coagulation in severe infections and effect of vitamin K1 intervention: a retrospective single-center analysis. Med Sci Monit. (2024) 30:e944778. doi: 10.12659/MSM.944778 39488729 PMC11542504

[13] Zaki HA Bensliman S Bashir K Iftikhar H Fayed MH Salem W . Accuracy of procalcitonin for diagnosing sepsis in adult patients admitted to the emergency department: a systematic review and meta-analysis. Syst Rev. (2024) 13:37. doi: 10.1186/s13643-023-02432-w, 38254218 PMC10802075

[14] Marra AR Lopes GO Pardo I Hsieh MK Kobayashi T Marra PS . Metagenomic next-generation sequencing in patients with fever of unknown origin: a comprehensive systematic literature review and meta-analysis. Diagn Microbiol Infect Dis. (2024) 110:116465. doi: 10.1016/j.diagmicrobio.2024.116465 39059148

[15] Chen Y Wang J Li M Niu T. Clinical and diagnostic values of metagenomic next-generation sequencing for infection in hematology patients: a systematic review and meta-analysis. BMC Infect Dis. (2024) 24:167. doi: 10.1186/s12879-024-09073-x, 38326763 PMC10848439

[16] Romano A Valluzzi RL Caruso C Dhruve H d’Ancona G Holmes S . Prescribing patterns and treatment adherence in patients with asthma during the COVID-19 pandemic. J Allergy Clin Immunol Pract. (2022) 10:100–107.e2. doi: 10.1016/j.jaip.2021.09.032, 34610490 PMC8487166

[17] Patel TS Nagel JL Newton M Oriel RC Shah A Anagnostou A . Food allergy management practices utilizing individual patient thresholds: a work group report of the AAAAI adverse reactions to foods committee. J Allergy Clin Immunol Pract. (2023) 11:1083–1086.e1. doi: 10.1016/j.jaip.2023.01.045, 36773718

[18] Liu X Yuan X Wen L Tan X Sui Q Liu J. Identification of risk factors and predictive indicators for tigecycline-associated hypofibrinogenemia. Clin Transl Sci. (2025) 18:e70213. doi: 10.1111/cts.70213, 40181433 PMC11968326

[19] Ma C Ren X Pang N Liu Y Chen M Zhang X . Incidence, characteristics, and risk factors of hypofibrinogenemia induced by generic tigecycline: a retrospective study. Naunyn Schmiedeberg's Arch Pharmacol. (2025) 398:2717–27. doi: 10.1007/s00210-024-03419-7, 39254879 PMC11919974

[20] Zhang L Wang H Liu M Ma H Gong Z Sun J . Development and validation of a risk prediction model for tigecycline-induced hypofibrinogenemia in septic patients: a retrospective cohort study. BMC Infect Dis. (2025) 25:683. doi: 10.1186/s12879-025-11019-w, 40340646 PMC12063261

[21] Li M He J Dong G Hu L Shao H. Serum concentration threshold and risk factors of tigecycline-induced hypofibrinogenaemia in critically ill patients. J Antimicrob Chemother. (2025) 80:200–8. doi: 10.1093/jac/dkae396, 39508368

[22] Paul M Shani V Muchtar E Kariv G Robenshtok E Leibovici L. Systematic review and meta-analysis of the efficacy of appropriate empiric antibiotic therapy for sepsis. Antimicrob Agents Chemother. (2010) 54:4851–4863. doi: 10.1128/AAC.00627-10 20733044 PMC2976147

[23] Tamma PD Aitken SL Bonomo RA Mathers AJ van Duin D Clancy CJ. Infectious Diseases Society of America 2023 guidance on the treatment of antimicrobial resistant gram-negative infections. Clin Infect Dis. (2023) 77:S313–22. doi: 10.1093/cid/ciad428, 37463564

